# A narrative review of neuro-ophthalmologic disease in African Americans and Hispanics with multiple sclerosis

**DOI:** 10.1177/20406223231202645

**Published:** 2023-09-30

**Authors:** Lauren Tardo, Amber Salter, Melanie Truong-Le, Lindsay Horton, Kyle M. Blackburn, Peter V. Sguigna

**Affiliations:** Department of Neurology, The University of Texas Southwestern Medical Center, 5323 Harry Hines Blvd, Dallas, TX 75390-8806, USA; Department of Neurology, The University of Texas Southwestern Medical Center, Dallas, TX, USA; Department of Neurology, The University of Texas Southwestern Medical Center, Dallas, TX, USA; Department of Ophthalmology, The University of Texas Southwestern Medical Center, Dallas, TX, USA; Department of Neurology, The University of Texas Southwestern Medical Center, Dallas, TX, USA; Department of Neurology, The University of Texas Southwestern Medical Center, Dallas, TX, USA; Department of Neurology, The University of Texas Southwestern Medical Center, Dallas, TX, USA

**Keywords:** African American, Hispanic, Latino, Latinx, multiple sclerosis, neuro-ophthalmology

## Abstract

Multiple sclerosis (MS) is the most common non-traumatic cause of disability in young people, with vision loss in the disease representing the second largest contributor to disability. In particular, African-American patients with MS are noted to have lower vision than their Caucasian counterparts. In this review, we examine the disparities in eye diseases in the MS population with our gaps in knowledge and discuss the underlying nature of pathological disparities.

## Introduction

Multiple sclerosis (MS) is a demyelinating disease of the central nervous system and is the most common cause of non-traumatic disability in young people.^1^ Disability accumulates by two separate components of the disease. Primarily, there is the relapsing form of the disease, where neurological symptoms often develop subacutely through inflammatory demyelinating plaques of the central nervous system with subsequent disability accumulation from incomplete recovery. Secondarily, there is an increased risk of neurodegeneration in a significant subpopulation of the disease, where neurologic disability accumulates independent of relapses, often termed the progressive form of the disease.^2^ Both forms of the disease can overlap and often lead to progressive neurological disability, often including vision loss.^3,4^

Epidemiologically, MS affects 2.8 million people in the world and approximately 1 million people in the United States.^5,6^ Historical literature in the field indicates that MS occurs more commonly in Caucasian patients; however, recent studies have demonstrated that the prevalence is higher than previously reported in African-American populations and possibly comparable when compared to Caucasian populations.^7–10^ The prevalence of MS among those who identify as Hispanic, Latino, and/or Latinx varies, but is lower than what is seen in African-American and Caucasian populations.^7,8^ However, this number is expected to grow as these populations now represent the most rapidly growing minority in the United States.^9^

While Hispanic, Latino, and/or Latinx are often used interchangeably when describing patient ethnicity, it should be noted that these terms have variable meanings. For this paper, we will refer to those who identify as Hispanic, Latino, and/or Latinx as Hispanics with MS (HwMS).^11,12^ In addition to rising numbers, it has also been well demonstrated that both African-American patients with MS (AAwMS) and HwMS have more aggressive disease courses and are more likely to suffer severe disability.^9,10,13^ Symptomatically, this disability accumulation manifests with a variety of neurological symptoms, including cognitive dysfunction, gait dysfunction, motor dysfunction, and sensory dysfunction; however, vision loss remains one of the most common symptoms of the disorder. There are some data to suggest that optic neuritis is a more common manifestation of the disease among both HwMS and AAwMS.^14^ In this narrative review, we summarize the neuro-ophthalmic manifestations in HwMS and AAwMS, inequities that are present in the literature, as well as underlying possible mechanisms.

## Body

### Optic neuritis

Broadly, optic neuritis is one of the most common neuro-ophthalmic diagnoses associated with MS. In approximately 20% of patients, optic neuritis is the initial presentation and is thought to occur in roughly 50% of patients in their lifetime.^4^ Often characterized by the subacute development of vision loss (decreased visual acuity with or without visual field loss), pain with eye movement, and dyschromatopsia color desaturation, inflammation and demyelination within the optic nerve cause delay of action potentials through the optic nerve, and secondary axonal block with eventual axon loss. While there is a strong association between MS and optic neuritis, there are a wide variety of etiologies to optic neuritis, with up to 31% of patients suffering from recurrent vision loss.^15^

Notably, given the association of optic neuritis and MS, the visual system has often been used as a window into the mechanisms of disease activity.^16^ Visual evoked potentials have provided a technology to measure the space constant of the optic nerves and have robust diagnostic and prognostic value.^17–19^ Optical coherence tomography (OCT) is another noninvasive technology that provides a great spatial resolution of the retina and has been used as a highly sensitive diagnostic evaluation for optic neuritis both in acute and chronic settings.^20–23^ Given the increasing precision of ophthalmic imaging technologies and increased availability, it is becoming an advantageous medium to detect pathological variability between minorities and non-minorities with MS.

Historically, differences between African-American and Caucasian patients with optic neuritis have been noted back to the optic neuritis treatment trial (ONTT).^24^ In this pivotal trial, there was no difference between sex, age, presence of disc edema, or fulfillment for MS at presentation between African Americans and Caucasians, but African-American patients had more frequent severe vision loss, both at presentation and at follow-up. This discrepancy was at least partially attributed to the increased prevalence of the ‘neuromyelitis optica’ presentation of neurological deficits, which occurred in 58% of the AAwMS studied. This hypothesis for the discrepancy remained as anti-aquaporin-4 (AQP4) antibody assays were not commercially available at the time of the study; however, anti-AQP4 was not detected in any of the 116 serological samples from the ONTT that were tested, making this hypothesis less likely.^25^ Over time, the differences in vision loss between AAwMS and Caucasians with multiple sclerosis (CAwMS) were felt to reflect the differentiation of risk for ‘opticospinal MS’ in African Americans relative to their Caucasian counterparts.^26^ As neuromyelitis optica spectrum disorder (NMOSD) became more differentiated from MS by the commercial availability and utilization of the anti-AQP4 assay, differences in vision loss between AAwMS and CAwMS became partially explained, but not fully accounted for. As serological positivity for the anti-AQP4 assay is now synonymous with NMOSD, a distinct demyelinating disease from MS, whether similar disparities exist for NMOSD remains an active area of research.

Not only did those AAwMS suffer from increased risk of disability accumulation through optic neuritis but also in global disability.^27^ This is perhaps partially explained by retinal neurodegeneration in the fellow, non-optic neuritis eye.^28,29^ Patients who identify as AAwMS and HwMS have been shown to have higher rates of disability when looking at the Patient-Derived Multiple Sclerosis Severity Score.^30^ There is frankly a dearth of neuro-ophthalmic data for HwMS, both in terms of specifics of structural and functional assessments of visual function.

Why AAwMS and HwMS suffer from increased visual disability and inflammatory disease activity is not fully understood. As historical data from MS suggest the disorder is underdiagnosed in racial and ethnic minorities, their disability data are not proportionally represented in the literature prior to recent efforts. Investigations into socioeconomic status have yielded some data to suggest an increased risk of disability accumulation; however, these covariates do not explain racial and/or ethnic disparities.^27,31^ Understanding the disparities between the neuro-ophthalmic findings in this population yields insight into the different pathological mechanisms.

### Microcystic macular edema

**Figure 1.**
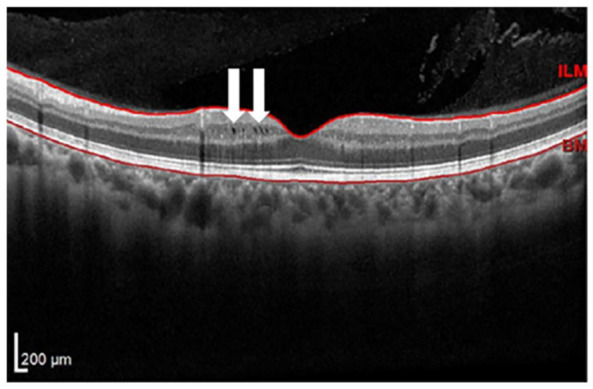
Example of incidentally noted microcystic macular edema within the inner nuclear layer in an AAwMS patient as part of screening for S1P modulator initiation. White arrows emphasize microscopic hyporeflective areas of edema. AAwMS, African-American patients with multiple sclerosis.

One glaring disparity in neuro-ophthalmic disease in MS is microcystic macular edema (MME). While technically a misnomer, MME is defined as cystic, lacunar areas of hyporeflectivity with clear boundaries within the inner nuclear layer (INL) of the retina, shown in Figure 1.^32^ MME could represent the microscopic precursor to macular edema, which in itself is associated with vision loss through a direct maculopathy.^33^ Although this direct comparison is controversial, longitudinal data do support this hypothetical spectrum of disease, although such data are limited. While MME is nonspecific, occurring in a multitude of disorders, it has a strong association with optic neuropathies such as optic neuritis and is well described in the MS literature.^34–36^

In the MS population, both MME and macular edema have been described. Of particular concern among AAwMS, the MME incidence is reported to be approximately 12% over a mean follow-up of 4 years, where overall frequency in the entire MS population is estimated to be anywhere from 1% to 6% over a slightly longer follow-up duration.^37^ Optic neuritis seems to be highly coincident with, but not sufficient, for the development of MME in MS. Interestingly, most cases of MME are transient, with only 16% remaining over a mean follow-up of 233 days in one study. There are some data to suggest that MME is associated with a higher risk of disease in MS patients, although it is less clear whether this is independent of race and/or ethnicity itself.^32^

The mechanism(s) of MME in MS is not precisely clear but were hypothesized to reflect dynamic fluid shifts, either through vascular permeability and/or Müller cell dysfunction, or perhaps non-inflammatory changes including mechanical traction or retrograde maculopathy.^38^ The inflammatory activity of optic neuritis being highly coincident in its development does suggest that inflammation, either chronic or acute, plays a significant role. Analogous to MS, MME is also strongly associated with NMOSD, where its mechanism was posited to involve direct insult to Müeller cells (which express AQP4, the primary immunogenic target of NMOSD) through a direct astrocytopathy.^39^ Interestingly, there are conflicting OCT data to support this hypothesis, perhaps again emphasizing multifocal mechanisms.^40–42^ While transsynaptic degeneration is increasingly appreciated in MS, a transsynaptic retrograde maculopathy is an unlikely explanation for its mechanism given the temporal dynamics of the disease.^43–45^ This, in combination with the lower prevalence of macular edema (1–6%) in contrast to highly prevalent neurosensory retinal loss appreciated with optic neuritis (60%), makes this similar at best a partial explanation.

Most pertinent in the therapeutic realm of MS was the early realization of an increased risk of MME following S1P modulation in patients with MS. The absolute risk of MME was low with fingolimod, the originally FDA-approved S1P modulator, at 1% annual incidence with exposure, with both diabetes and uveitis identified as risk factors for its development.^45^ These risk factors for S1P-associated MME furthered the hypothesis that vascular permeability may be playing a significant role in the pathophysiology of MME for most MS patients. As such, screening for macular edema and MME became integral to S1P prescription, as some patients have developed the adverse event 24 h following therapy initiation.^46^ Given the high prevalence of MME noted in AAwMS, initiation of S1P modulation should follow local prescribing regulations, with careful consideration of MME risk, as this finding is transitory in the majority of cases. Whether those with a history of MME that has resolved are at risk of MME and/or macular edema following S1P modulation has not been examined.

The mechanism of MME as an adverse of S1P modulation is similarly unclear. It was originally hypothesized that S1P3 modulation at the orbital vascular endothelial junctions was directly responsible, although macular edema has been reported as an adverse event with ponesimod, a highly selective S1P1 modulator.^47,48^ In addition, MME has been encountered in S1P modulation in other non-neurological conditions, such as ulcerative colitis, which argues against a direct neuroinflammatory effect underlying its pathogenesis. There are data to suggest that nonselective S1P modulation may be associated with an increase in macular volume, which could represent subclinical macular edema, although this hypothesis was limited by the single-center design, and did not report measures of the INL, where the majority of MME presents.^49^ Despite the risk of macular edema associated with S1P modulators, a systematic review indicated that a favorable outcome was observed in select cases with continuation of this therapy with concomitant macular edema treatment when alternative therapies were untenable, although extreme caution is highly encouraged in such cases.

**Figure 2.**
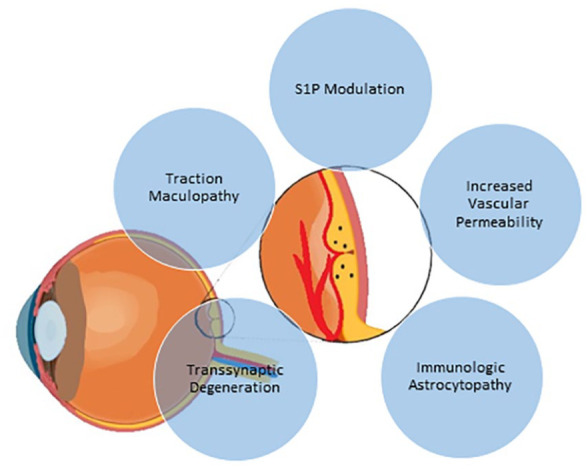
Theoretical mechanisms of microcystic macular edema in patients with multiple sclerosis.

While MME represents a relatively uncommon neuro-ophthalmic finding in MS, it has significant implications both diagnostically and therapeutically for most patients with MS. A variety of mechanisms have been posited to explain its mechanism(s), shown diagrammatically in Figure 2. With available data to date, the increased prevalence of MME in AAwMS possibly reflects either immunologic or vascular risk factors. Further studies are needed to clarify the precise mechanisms of this disparity to reduce the risk of vision loss.

### Retinal neurodegeneration

As MS has both an inflammatory and neurodegenerative component, the visual system has been used to capture quantitative measures of disease activity. Disease activity was primarily appreciated through disease within the optic nerve itself. The retinal nerve fiber layer thickness, an OCT metric often taken as a surrogate for axons within the optic nerve, has captured neurosensory retinal loss, known to occur in patients with MS at an average rate of 0.4 µm/year, whereas healthy controls lose on average 0.2 µm/year.^37^ Indeed, neurosensory retinal loss measured in a variety of ways is accelerated in those who identify as African American relative to Caucasian, primarily within the inner retina.^27^ In addition, macular volume loss correlates well with global brain volume loss, serum neurofilament light chain levels,^50,51^ as well as with visual function, including low contrast visual acuity.^52–54^ Taken cumulatively, there are data to suggest the cross-sectional volume of the macula has significant prognosis value, with lower macular volumes being predictive of an increased risk of disability accumulation at 10 years, regardless of optic neuritis.^55^ In this way, measurement of retinal structure and function captures ‘silent progression’ in MS, what is globally being described as progression independent of relapse activity.^56^

Analogous to brain volumetric studies, it is known that several variables, such as inflammatory disease activity and obesity, can affect neurosensory retinal loss.^57,58^ In terms of health disparities, there is evidence that obesity may have differential effects in racial subgroups. Investigation into the socioeconomic subgroups independent of race has shown a similar relationship of higher neurosensory retinal loss with lower socioeconomic status measured by a variety of variables, including median household income, education level, and state area deprivation index.^24,52^

As the mechanisms of disability accumulation in MS are multifactorial, further study into the dynamics of both structure and function through the visual system yields the opportunity to understand the disease in a more diverse patient population. Indeed, as OCT-based diagnostic criteria are considered potential diagnostics criteria for MS, incorporating ethnically and racially diverse patient cohorts will be critical to their generalization.^22^ The quantitative methodologies available to neuro-ophthalmology make the incorporation of immunological, environmental, and socioeconomic covariates into disease models of disability ultimately highly tenable.

### Genetics

While the pathogenesis of MS is complex, it has been well documented that genetics play a role in the development of MS, with the class II region of human leukocyte antigen (HLA) having the largest effect, in addition to a growing number of almost 200 other genetic loci.^59,60^ Studies evaluating the specific roles in AAwMS have demonstrated that there is a difference in these previously established genetic risk variants among AAwMS with both HLA-DRB1*15:01 and HLA-DRB1*15:03 inferring an increased risk of developing MS consistent with other populations, but HLA-DQB1*06:02 having no association with increased risk.^61,62^ Within certain Latin American populations, HLA-DRB1*15:01, HLA-DRB1*15:03, and HLA-DQB1*06:02 have shown an association with increased risk similar to what is seen in other populations.^63^ Studies have also demonstrated that there is a genetic influence on the age of onset in HwMS leading to MS presenting at earlier ages; however, there is conflicting data surrounding this idea in AAwMS.^8^

More interesting and specific to this current narrative review, there appears to be a genetic influence on the development of optic neuritis as a presenting symptom in MS among Hispanics based on genetic admixture, the interbreeding of individuals from isolated lineages.^64^ In a 2018 study, Amezcua *et al.* demonstrated regional differences in the development of optic neuritis in HwMS, with those with Native American ancestry in the southwestern United States having higher rates of optic neuritis relative to CAwMS.^14,65^ Similar studies have not been replicated in AAwMS.

### Mechanisms

The answer to why there is an interethnic and interracial difference in disease aggressiveness is likely complex, with influence from genetics, environmental exposures, immunological variations, as well as social determinants of health. Specific to immunological mechanisms, one possible explanation relates to the differing pathological phenotypes of MS. The four classic pathologically determined lesion types, from type I to type IV, are based on different immune system characteristics. Of specific relevance are type II lesions, which are characterized by antibody deposition, and thus directly implicating the humoral immune system in the development of MS lesions.^66^ The humoral immune system is highly active in both AAwMS and HwMS, as there are increased levels of antibody-secreting cells in peripheral blood samples.^67^ Furthermore, it is now known that AAwMS have higher levels of intrathecal humoral inflammatory changes based on higher levels of IgG synthesis rate and IgG index.^68^ It has also been demonstrated that the presence of oligoclonal bands is associated with higher levels of disability among this patient population, and so one possible explanation for the disparate disability could relate to this immunological heterogeneity.^69,70^ There is further anatomic evidence to support this hypothesis, as there is also an association with cerebral gray matter volume loss and the presence of an elevated cerebrospinal fluid (CSF) IgG index in AAwMS.^71^

Another argument for an immunological disparity can be made by exploring the interracial data on exposure to disease-modifying therapies (DMTs), as AAwMS have been shown to have a less robust therapeutic response to certain DMTs. *Post hoc* analysis of the EVIDENCE trial, which evaluated the efficacy of interferon-β-1a in treatment-naïve MS patients, demonstrated that AAwMS treated with interferon-β-1a suffered from a higher number of exacerbations were more likely to have a relapse, and developed a higher number of new T2 lesions at 48 weeks compared to CAwMS.^72^ This differing treatment response was further illustrated in a retrospective review comparing treatment response to interferon-β-1a or interferon-β-1b, glatiramer acetate, as well as natalizumab in AAwMS compared to CAwMS suggesting this relationship is not DMT specific among the early DMTs.^73^ There is currently very limited data evaluating DMT exposure in HwMS, making analogous comparisons untenable.

### Uveitis

Historically, there has long been an appreciated relationship between uveitis and MS, where the incidence varied between 10% and 23%.^74,75^ While early data identified that those with African ethnicity are more likely to have these uveitis, in recent literature, the prevalence of uveitis in MS has decreased to 0.51%.^76^ In addition, in the large UK Biobank study, 87.5% with both MS and uveitis identified as White. In the modern era, given the relatively low incidence of uveitis, it is difficult to understand the contribution of both race and ethnicity to this disease pathophysiology. In addition, the uveitis is often anterior or intermediate, for these reasons, is beyond the scope of this narrative review.

### Ocular dysmotility

Similarly, ocular dysmotility, including internuclear ophthalmoplegia (INO) and wall-eyed bilateral INO, are highly prevalent in MS.^77^ There is a dearth of literature examining ethnic and racial disparities in these findings, which is worth mentioning, particularly as there is literature suggesting that structurally the brainstem regions controlling these functions are selectively vulnerable in those with African-American ancestry.^78^ Further study is needed on this topic.

### Disease-modifying therapies

Despite the increased prevalence and higher risk for aggressive disease in AAwMS and HwMS, both populations are grossly underrepresented in research efforts. A PubMed review completed in 2014 by Khan *et al.*^13^ found that less than 1% of the 60,000 publications on MS were focused on minority populations.^13^ The lack of minority representation in clinical trials is even more concerning, with a recent review highlighting that less than 10% of patients in these trials were representative of minority backgrounds.^79^ As mentioned previously, it has been shown that AAwMS have less robust responses to early DMTs, but whether this relationship continues among the more recent DMTs remains unknown. Furthermore, similar relationships are not known to exist between HwMS and CAwMS.

Given the increasing attention to representation disparities, there have been more recent efforts to bridge this gap. The CHIMES trial is a phase IV clinical trial evaluating the efficacy and safety of ocrelizumab in minority patients with relapsing forms of MS. The findings from this trial will broaden our knowledge of disparate pathology among AAwMS and HwMS.^80^ In addition, the results of the FLUENT study, which investigated the change in peripheral lymphocyte dynamics in patients with relapsing forms of MS treated with fingolimod, demonstrated a reduction in both T cells (CD4^+^ and CD8^+^) and B cells (CD19^+^ naïve and memory) in a patient treated with fingolimod.^81^ While not directly aimed at evaluating treatment responses in minority MS patient populations, these results highlight that the S1P receptor modulator class of medications could be particularly beneficial in AAwMS given the higher level of B-cell activity noted in the peripheral blood of these patients.^82^ Ultimately, we must continue to broaden our understanding of DMT use in these patient populations to have a better understanding as to why these patient populations accumulate greater visual and global disability.

## Conclusion

**Figure 3.**
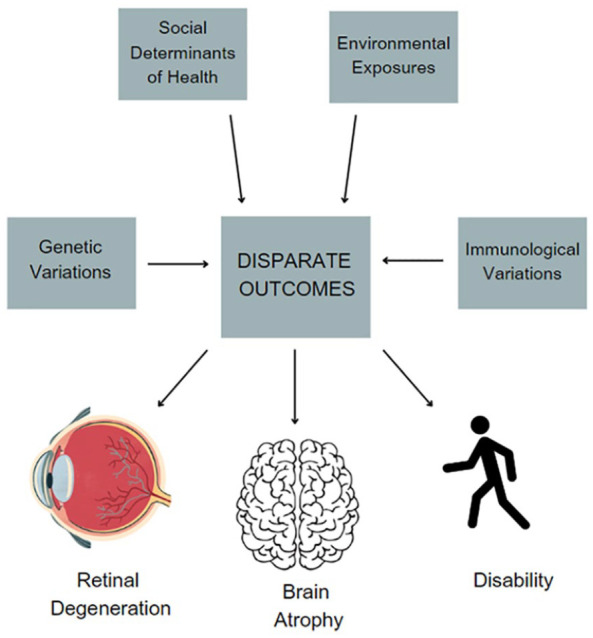
Model of influences regarding disparate outcomes in multiple sclerosis. Source: Created with canva.com.

While MS is the most disabling non-traumatic cause of young people, there are disparities and inequities that require both scientific and social attention. There are a number of lines of evidence that AAwMS and HwMS are at increased risk of disability accumulation. This can be seen in markers of clinical disability, radiographic outcomes, and visual outcomes, shown diagrammatically in Figure 3. Here, we took a deep dive into the mechanisms of heterogeneous outcomes surrounding vision loss where the mechanisms likely reflect an increased risk of both inflammatory and degenerative processes, which are often difficult clinically to disentangle. The mechanisms of this vision loss likely reflect an increased risk of both inflammatory and degenerative processes, which are often difficult clinically to disentangle. In particular, while the mechanisms of progressive MS remain an area of debate, there is evidence that neuro-ophthalmic studies may provide the much-needed window into the cellular and bioenergetic mechanisms of the disease to further disentangle mechanisms and potential therapies.

Lastly, while there are relationships between the neuro-ophthalmic findings among AAwMS relative to CAwMS, analogous data for HwMS are desperately needed. As this represents a binarization of a variable of interest, a robust relation is likely subject to both type I and type II errors. If interethnic neuro-ophthalmic disease supports a similar disparity, this may provide the data needed for proposed universal mechanisms, and garner a greater understanding of how their participation in future therapy development will affect outcomes.

This review has a number of limitations. First and foremost is its narrative format. There is both diverse and comprehensive literature on disparities in MS, and so the narrative format permits the appreciation of a number of topics, but should not be considered exhaustive in its coverage. The authors refer the reader to a number of the references for additional reading.

## Declarations

**Ethics approval and consent to participate:** Not applicable.

**Consent for publication:** Not applicable.

**Availability of data and materials:** Not applicable.
